# Nonstandard usage of a left ventricular assist device in a patient with severe heart failure complicated by pulmonary artery thrombosis - case report

**DOI:** 10.1186/s13019-020-01169-0

**Published:** 2020-06-03

**Authors:** Jerzy Pacholewicz, Szymon Leonik, Maciej Sojka, Paweł Nadziakiewicz

**Affiliations:** 1 Department of Cardiac, Vascular and Endovascular Surgery and Transplantology, Medical University of Silesia in Katowice, Silesian Centre for Heart Diseases, Zabrze, Poland; 2 Department of Cardioanesthesia and Intensive Therapy, Medical University of Silesia in Katowice, Silesian Centre for Heart Diseases, Zabrze, Poland

**Keywords:** Heart failure, Continuous flow assist device, Dilated cardiomyopathy, Pulmonary embolism, Case report

## Abstract

**Background:**

Heart failure complicated by pulmonary embolism is an extremely rare condition described in the literature. We report a case of very young patient with advanced heart failure against the background of dilated cardiomyopathy of unknown etiology with the presence of blood clots in both ventricles.

**Case presentation:**

The course of treatment was complicated by acute pulmonary embolism. In emergency setting the patient was qualified for combine surgery pulmonary embolization and implantation of a continuous flow pump as a bridge for heart transplantation. The post-operative course is described in detail as well as reimplantation of the pump due to early thrombosis.

**Conclusions:**

Performed surgical procedures combined with alteration in anticoagulant drugs was sufficient to stabilize the clinical condition.

## Introduction

Despite considerable advances in the treatment of severe heart failure, long-term survival is poor. Therapeutic schemes are effective for most clinical cases. However, some of them require non-standard procedures.

## Case report

**Fig. 1:**
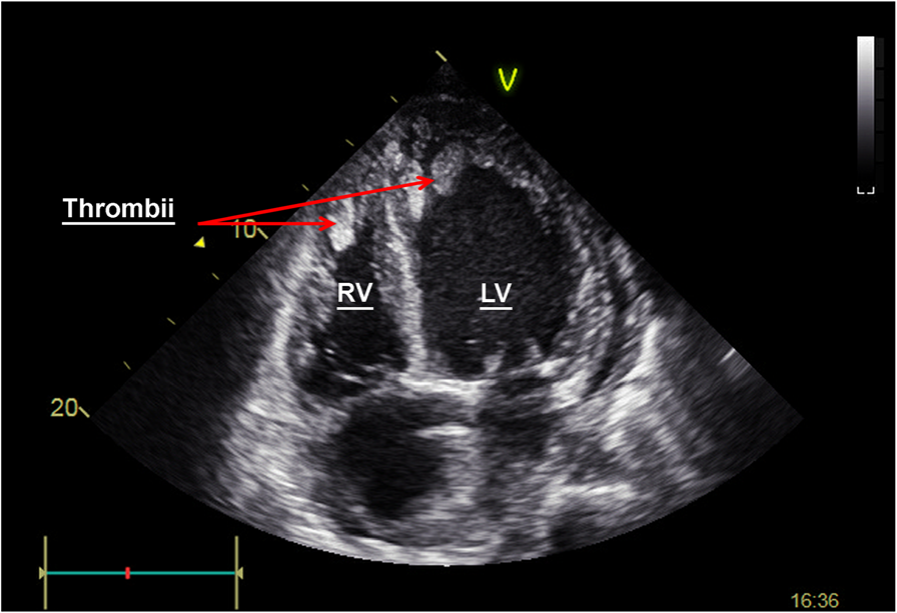
Trombus formation in left and right ventricle in echocardiography. RVOT - right ventricular outflow tract,, LV – left ventricle

**Fig. 2:**
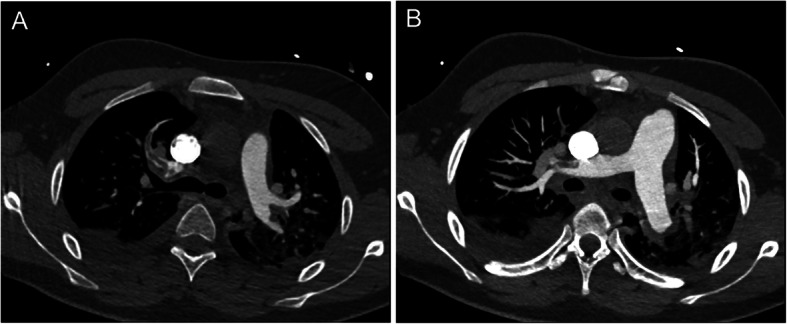
The saddle embolism in the lumen of the right upper lobar artery (**a** and **b**)

An 18-year-old patient was admitted to the ward with severe heart failure with a background of dilated cardiomyopathy of unknown aetiology. On admission day he was hemodynamically stable, however, during the night patient developed dyspnoea accompanied by haemodynamic instability and cardiogenic shock. In the echocardiography, two mobile thrombi of 20x10mm were visualised in both ventricles (Fig. 1). The bases of the thrombi were attached to the endocardium. The left ventricular ejection fraction was 18%. Additionally, a pulmonary embolism was revealed in the computed tomography scan (Fig. 2). Considering the Wells scale (original version), the patient obtained < 4 points. In laboratory tests there were no significant signs of coagulation disorders besides mild fibrinogen elevation to 420 mg/dl and 1.99 μg/ml of D-dimer. The D-dimer elevation would indicate the forming of a thrombus, however, the fibrinogen levels suggests it was of a low intensity.

Regarding the patient’s deteriorating condition, a decision was made to implant, a left ventricular assist device (LVAD) as a bridge to transplantation. The removal of the thrombus from the left ventricle and a pulmonary embolectomy were performed simultaneously.

Two days after implantation, a more than two-fold pump power consumption increase and a lactate dehydrogenase concentration exceeding the norm (up to 5775 IU/l) was observed. This suggested haemolysis within the implanted LVAD. Thrombosis occurred despite the implementation of anticoagulant therapy. In regard to the postoperative condition, it wasn’t possible to apply thrombolytic therapy. Intravenous, anticoagulant heparin treatment proved to be ineffective. Considering the deteriorating condition and confirmation of active coagulation within the device by echocardiography, the decision was made to urgently substitute the LVAD.

After reoperation, paroxysmal atrial fibrillation was observed, which was controlled with intravenous amiodarone. The symptoms of a respiratory tract infection required microbiological diagnosis. In the sputum culture, abundant growth of *Klebsiella pneumoniae* ESBL (+) and *Candida dubliniensis* was detected. Due to postoperative acute renal failure and anuria, haemodiafiltration was carried out. These events had a negative impact on the patient’s condition and extended his rehabilitation. During the hospitalisation, an autoimmune disease was suspected. The suspicion was initially confirmed by the positive result of lupus anticoagulant (1.3, reference value > 1.2). However, negative results of anti-nuclear antibodies, anti-neutrophil cytoplasmic antibodies and other characteristic for lupus antibodies may suggest remission or absence of the ailment. The presence of anti-DFS70 was also not demonstrated. In the presence of this antibody, the chance of rheumatic or autoimmune diseases is lower. With regard to the full picture, we couldn’t exclude or confirm an autoimmune disease.

## Discussion

Heart failure complicated by pulmonary embolism is an extremely rare condition described in the literature. In the following case, it is reasonable to perform the procedures of simultaneous removal of thrombi and LVAD implantation. In the group of young patients affected by pulmonary embolism it seems reasonable to undertake an in-depth diagnosis. An aspect worth considering in the context of distant therapy, is the possible impact of the genetic load on a predisposition to further thromboembolic complications. An important factor in the prevention of early cardiovascular disease is the control of systemic lupus erythematosus, which significantly changes the dynamic of the endovascular endometrium and may worsen the prognosis [1–3].

## Conclusion

Long-term VAD treatment was effective in this patient with severe heart failure, despite the ventricular thrombosis and acute pulmonary embolisation. In our experience, the combined surgical procedure of implantation of a left ventricular assist device and a pulmonary embolectomy was the only possible way to save the patient’s life. The infection in the respiratory system and the suspected autoimmune disease additionally complicated the course of acute heart failure due to the presence of thrombus formation in the ventricular cavities.

## Data Availability

Not applicable. The datasets analyzed during the study are available from the corresponding author on reasonable request.

## Abbreviations

LVAD: Left ventricular assist device
